# Sweroside Inhibits Inflammation and Alleviates Endothelial Injury and Atherosclerosis in Mice

**DOI:** 10.1111/jcmm.70837

**Published:** 2025-09-05

**Authors:** Minjiang Huang, Huiming Yin, Qiansheng Yang, Lingli Liu, Yuefu Chen, Ling Jin, Yang Yang, Ke Hu, Yan Ding

**Affiliations:** ^1^ Department of Diagnostics Hunan University of Medicine Huaihua Hunan China; ^2^ Department of Respiratory and Critical Care Medicine, First Affiliated Hospital Hunan University of Medicine Huaihua Hunan China; ^3^ School of Pharmaceutical Sciences Hunan University of Medicine Huaihua Hunan China

**Keywords:** atherosclerosis, inflammation, sweroside

## Abstract

The underlying mechanisms in atherosclerotic vascular diseases are not entirely clear, posing a challenging hurdle to treatment. Inflammation is a root cause of atherosclerosis (AS); therefore, anti‐inflammatory agents have potential for its management. Sweroside, possessing anti‐inflammatory properties, emerges as a potential agent to impede AS progression. In this study, we investigated the effects of sweroside on AS mice and elucidated its molecular mechanisms. We conducted in vivo experiments using an apolipoprotein E mice model of AS to explore the effects of sweroside on vascular inflammation adhesion responses, endothelial injury and AS. In vitro experiments, mouse aorta endothelial cells were treated with palmitic acid (PA) and sweroside, and the protective effects of sweroside on endothelial injury were analysed. AS is a chronic inflammatory disease and activation of nuclear factor κB (NF‐κB) signalling contributes to inflammatory reactions and AS. Mitogen‐activated protein kinase kinase kinase kinase 4 (MAP4K4) has been identified as an upstream target of NF‐κB signalling. We detected MAP4K4/NF‐κB signalling pathways using gene siRNA silencing and knockdown assays and investigated the protective effects of sweroside in PA‐mediated endothelial injury and western‐diet‐induced AS. The findings demonstrated that sweroside attenuated vascular inflammation, adhesion responses, and leukocyte homing and alleviated endothelial injury and atherosclerosis in vivo. Sweroside attenuated endothelial inflammation, apoptosis, permeability and adhesion responses induced by PA in vitro. Sweroside alleviated endothelial injury and atherosclerosis through MAP4K4/NF‐κB signalling. Hence, sweroside is a promising candidate for treating AS, acting by targeting the MAP4K4/NF‐κB pathway.

## 1 Introduction

Atherosclerosis (AS) is a highly prevalent vascular disease in older adults with a high fatality rate that detrimentally impacts quality of life [1, 2]. It is well known that chronic vascular inflammation plays a key role in the development of AS by increasing the abundance of cell surface adhesion molecules, thus exacerbating vascular injury [3, 4, 5]. AS is a chronic inflammatory disease. Activation of nuclear factor κB (NF‐κB) contributes to inflammatory reactions, and endothelial‐cell‐specific NF‐κB inhibition protects mice from AS [6]. Endothelial NF‐κB is essential for the expression of leukocyte adhesion molecules, AS, and macrophage homing to aortic plaques [6, 7, 8]. Mitogen‐activated protein kinase kinase kinase kinase 4 (MAP4K4) has been identified as an upstream regulator of NF‐κB signalling, which plays a key role in regulating the development of AS [5]. Anti‐inflammatory drugs can potentially protect the endothelium against AS‐induced injury [9]. Nevertheless, most anti‐inflammatory drugs effectiveness is limited, and the accompanying risk of complications is considerable. Thus, exploring intervention strategies with significant anti‐inflammatory effects and low toxic side effects is needed.

Sweroside is a common iridoid with antioxidant and anti‐inflammatory properties [10, 11]. Several studies have suggested that sweroside exhibits therapeutic effects in various inflammatory diseases, such as liver injury, nonalcoholic fatty liver disease and diabetic nephropathy [12, 13, 14]. Hence, sweroside appears to hold therapeutic potential for AS by inhibiting the initial inflammatory stage. Our previous investigation demonstrated that inhibiting MAP4K4/NF‐κB signalling pathways in endothelial cells can prevent the development of AS, presenting MAP4K4/NF‐κB as a crucial therapeutic target [15]. However, the question of whether sweroside can mitigate or prevent inflammation‐induced AS by targeting potential MAP4K4/NF‐κB signalling pathways requires further exploration.

This study aimed to assess the effect of sweroside in the treatment of inflammation‐induced AS and to elucidate its possible underlying mechanisms. We hypothesised that sweroside could alleviate endothelial injury and AS through inhibiting the MAP4K4/NF‐κB pathway. In this study, mouse aortic endothelial cells (MAECs) and gene‐edited mice with MAP4K4 knockdown were used to validate the above hypothesis and provide initial insights into the molecular mechanism underlying the anti‐inflammatory effect of sweroside.

## 2 Materials and Methods

### 2.1 Sweroside Preparation

Sweroside was purchased from Sigma‐Aldrich (St. Louis, MO, USA) and stored at 4°C before use. The concentration of the stock solution of sweroside in dimethyl sulfoxide (DMSO) was 10 mg/mL, which was diluted with phosphate buffered saline (PBS) for use. In cell experiments, the control group was treated with 0.16% PBS‐DMSO.

### 2.2 Animals and Treatments

Wild‐type (WT) C57BL/6J and apolipoprotein E knockout (AKO) mice were purchased from Shanghai Southern Model Biotechnology Co. Ltd. (Shanghai, China). MAP4K4‐pSico mice were generated by a lentiviral vector as previously described [5], and then crossed with VE‐cadherin Cre transgenic mice to generate mice with endothelial‐specific knockdown (KD) of MAP4K4. All mice were housed in a pathogen‐free environment under a 12‐h light–dark cycle at a constant temperature of 23°C ± 1°C with free access to food and water. From 4 to 6 weeks of age, the mice were fed a normal chow diet (NCD) or western diet (WD) (40% kcal fat, 43% kcal carbohydrates and 17% kcal protein; Beijing HFK Bioscience Co. Ltd., Beijing, China) for 12 weeks.

The mice were divided into a control group (WT‐Con), a control + sweroside group (WT‐Sweroside), an AKO group (AKO‐Con) and an AKO + sweroside group (AKO‐Sweroside) (*n* = 10 per group). In the above experiments, WT‐Sweroside and AKO‐Sweroside mice were administered 120 mg/kg/day sweroside by gavage for 3 months. The dosage was determined to be reasonable and non‐toxic. The other two groups of mice were administered saline intragastrically with the same amount saline for three months. The animals were sacrificed with 1% sodium pentobarbital for tissue extraction. All animal procedures were approved by the Animal Ethics Committee of Hunan University of Medicine (approval no. 2022(A03003)) and conducted in accordance with the Guide for the Care and Use of Laboratory Animals.

### 2.3 Generation of Mice

The original pSico‐MAP4K4 lentiviral vector was constructed as described previously [5]. A conditional U6 promoter was created by inserting a cytomegalovirus (CMV)‐enhanced stop cassette between two loxP sites. Thus, a functional U6 promoter was obtained after Cre excision to drive expression of a short hairpin RNA (shRNA) targeting MAP4K4 (5′‐GCT GTC TGG TGA AGA ATT A‐3′). The poly(A) tail required for CMV‐enhanced expression of green fluorescent protein (GFP) was located in the 3′ self‐inactivating long terminal repeat to exclude the possibility of potential interference of enhanced GFP (EGFP) expression in primary tissues. Small interfering RNA (siRNA) targeting MAP4K4 transcripts was produced by the U6 promoter, as the 6‐nucleotide poly(T) sequence at the end of the MAP4K4 shRNA antisense sequence is recognised as a termination signal by RNA pol III promoters, including U6. Then, the construct was injected into eggs at the one‐cell stage and two‐cell stage eggs were implanted into female pseudo‐pregnant C57BL6/J mice. Next, the mice were bred with C57BL6/J mice for seven generations. Genomic DNA was extracted from the obtained mice and subjected to polymerase chain reaction (PCR) for genotyping (shRNA primers: 5′‐CCC GTA TGG CTT TCA TTT TCT CC‐3′ and 5′‐AAG GAA GGT CCG CTG GAT TGA G‐3′).

### 2.4 Tissue Collection and Histological Analysis

Aortas were removed from mice, and atherosclerotic plaque was collected from the whole aortas and cross‐sections of the aortic root and then stained with Oil Red O (sigma, USA) [15]. Immunohistochemical staining was performed using standard procedures as previously described [16]. Sections of the 20 μm aorta plaque were deparaffinised, rehydrated and incubated overnight at 4°C with a polyclonal antibody (Ab) against CD68 (dilution, 1:200; Boster Biological Technology, Pleasanton, CA, USA, BA3638), followed by incubation with horseradish peroxidase‐conjugated anti‐immunoglobulin secondary Abs (dilution, 1:200; Jackson Immuno Research Laboratories, West Grove, USA) and visualised with 3, 3′‐diaminobenzidine. The slides were counterstained with haematoxylin. The area of positive staining was measured in five different fields of each slide at 400× magnification and quantified using Image‐Pro Plus 6.0 software (Media Cybernetics, Rockville, MD, USA).

### 2.5 Analysis of Endothelial Cells (ECs) Apoptosis In Vivo

As described in a previous report [17], apoptosis of ECs in thoracic aortas was detected by double staining with an Ab against Cleaved Caspase‐3 (dilution, 1:100; Cell Signalling Technology, USA, #9661) and a monoclonal Ab against CD31 (dilution, 1:100; ABclonal Technology, ab24590). Finally, ultrathin sections of the thoracic segments were examined with a transmission electron microscope (HT7700; Hitachi High‐Technologies Corporation, Tokyo, Japan).

### 2.6 Leukocyte Homing Assay

The leukocyte homing assay was performed as described previously [5]. Peritoneal exudate cells of male C57BL/6‐Tg (CAG‐EGFP)1Osb/J mice (age, 8 weeks; The Jackson Laboratory) were stimulated by intraperitoneal injection of 4% thioglycolate. After 2 days, 3 million cells were injected intravenously into mice in the AKO + saline control (AKO‐Con) and AKO + Sweroside groups fed a WD for 12 weeks. After 2 days, the aortic roots were harvested and embedded in optimal cutting temperature compound. The total number of fluorescent cells was counted in 10 sections (8 mm) over a 0.5‐mm area and normalised to the plaque area.

### 2.7 Glucose and Insulin Tolerance Tests and Biochemical Assays

The glucose and insulin tolerance tests were performed as described previously [18]. Blood samples were obtained from the tail veins of mice after a 12‐h fast and stored at −80°C until assayed. Blood glucose was measured using a blood glucose meter (Bayer Healthcare AG, Leverkusen, Germany). Serum levels of insulin, glycated haemoglobin (HbA1c), tumour necrosis factor (TNF)‐α, interleukin (IL)‐6, IL‐1β, intercellular adhesion molecule 1 (ICAM‐1), vascular cell adhesion protein 1 (VCAM‐1) and E‐selectin were measured using enzyme‐linked immunosorbent assay (ELISA) kits (R&D Systems Inc., Minneapolis, MN, USA). Serum levels of high‐density lipoprotein cholesterol (HDL‐C), low‐density lipoprotein cholesterol (LDL‐C), triglyceride (TG), total cholesterol (TC) and free fatty acids (FFA) were measured using commercial colourimetric assay kits (Nanjing Jiancheng Bioengineering Institute, Nanjing, China) in accordance with the manufacturer's instructions.

### 2.8 Isolation and Culture of Primary Mouse Aortic Endothelial Cells

The primary MAECs were isolated as described in a previous report [16, 19]. First, the aorta was dissected out from the aortic arch to the abdominal aorta and immersed in 20% fetal bovine serum (FBS)‐M199 medium containing 10 mg/mL of heparin. A 24‐gauge cannula was inserted into the proximal portion of the aorta. After ligation with a silk thread at the site, the inside of the lumen was briefly washed with serum‐free M199 medium. The other side was sealed and filled with collagenase type II solution (2 mg/mL, dissolved in serum‐free M199 medium). After incubation for 30 min at 37°C, ECs were removed from the aorta by flushing with 5 mL of M199 medium containing 20% FBS. The ECs were collected by centrifugation at 1200 rpm for 5 min. Then the precipitate was gently resuspended in 2 mL of 20% FBS‐M199 medium and cultured in a 35 mm collagen Type I‐coated dish. After 2 h incubation at 37°C, the medium was removed, the cells were washed with warmed PBS, and medium G was added. One week later, confluent ECs were observable. The cells were selected with anti‐rat magnetic Dynabeads (Life Technologies, Carlsbad, CA, USA) conjugated to monoclonal Abs (Abcam, Cambridge, MA, USA) against CD31 (ab124432) and CD102 (ab231564), and cultured in M199 medium (Sigma‐Aldrich Corporation, St. Louis, MO, USA) supplemented with 20% FBS (Gibco; Thermo Fisher Corporation, Carlsbad, CA, USA), heparin (10 mg/mL) and EC growth factor (50 μg/mL) at 37°C under a humidified atmosphere of 5% CO_2_/95% air to confluence. Unselected cells, as the nonendothelial fraction, were cultured to confluence. MAECs were confirmed by fluorescent staining of Abs against von Willebrand factor. MAECs at passages 3 to 6 were used for all experiments.

### 2.9 Apoptosis of ECs and Endothelial Permeability In Vitro

MAECs were pretreated with or without 1 μg/mL of sweroside for 24 h, followed by additional treatment with PA (0.4 mM for 24 h; Sigma‐Aldrich Corporation) when needed. The proportion of apoptotic cells was determined by flow cytometry with an Annexin V‐fluorescein isothiocyanate/propidium iodide assay (BD Biosciences, San Jose, CA, USA) in accordance with the manufacturer's instructions. Endothelial permeability was assessed with an in vitro vascular permeability assay kit (EMD Millipore Corporation, Billerica, MA, USA) in accordance with the manufacturer's instructions.

### 2.10 Cell Migration Assay

Migration of MAECs was analysed with the Transwell assay using a modified Boyden chamber (pore size, 8 μm) (BD Biosciences). MAECs (~10^5^) were added to the upper compartment of the transwell chamber, and sweroside for 24 h or PA for 24 h was added to the lower chamber. Cell migration in five random high‐power fields (×400) was assessed with a light microscope after staining with Crystal Violet dye.

### 2.11 Silencing of MAP4K4 in MAECs

Silencing of MAP4K4 in MAECs was conducted as described in a previous report [15]. Briefly, MAECs were transfected with siRNA targeting MAP4K4 (siMAP4K4) or a control (scrambled) siRNA (siCon) using Lipofectamine RNAiMAX Transfection Reagent (Invitrogen Corporation) in Opti‐MEM medium (Invitrogen Corporation) in accordance with the manufacturer's instructions. The transfection efficiency of MAECs reached 80%. The siRNA sequences are listed in Table S1.

### 2.12 Reverse Transcription‐Polymerase Chain Reaction (RT‐PCR) Analysis

RT‐PCR analysis was performed as previously described [15]. The thermal cycling conditions consisted of an initial denaturation step at 95°C for 3 min, followed by 40 cycles at 95°C for 5 s, 55°C for 15 s and at 72°C for 10 s. The expression levels of the genes of interest were normalised to the relative expression level of GAPDH. The primer sequences used for RT‐PCR analysis are listed in Table S1.

### 2.13 Cell Reactive Oxygen Species (ROS) Staining Assay

MAECs were pretreated with or without 1 μg/mL of sweroside for 24 h, followed by additional treatment with 0.4 mM PA for 24 h when needed. ROS levels were measured using a ROS assay kit (Beyotime, S0033S‐1) according to the manufacturer's protocol. Briefly, cells were washed twice with PBS and incubated with 10 μmol/L DCFH‐DA (diluted 1:1000 in serum‐free medium) at 37°C for 20 min. After washing, cells were fixed with 4% paraformaldehyde and mounted for imaging. Green fluorescence (indicative of ROS) was visualised under a fluorescence microscope (Nikon, ECLIPSE Ts2). Images were analysed using ImageJ software to quantify fluorescence intensity.

### 2.14 Immunofluorescence Staining

Immunofluorescence staining was performed using a monoclonal Ab against p65 (dilution, 1:400; Cell Signalling Technology Inc. #8242). Photomicrographs were acquired with a laser confocal microscope (Carl Zeiss AG, Jena, Germany) and analysed with ImageJ software.

### 2.15 Western Blot Analysis

The collection and lysate of MAECs and/or cell extracts in RIPA buffer (Millipore, Bedford, MA), but the buffer does not contain protease and phosphatase inhibitors, so they were incubated on ice for 30 min. Proteins were separated by gel electrophoresis and transferred onto nitrocellulose membranes (Millipore). The incubation of membranes with the primary antibodies: phosphorylated c‐Jun N‐terminal kinase (JNK) (p)‐JNK (Thr183/Tyr185) (dilution, 1:2000; Cell Signalling Technology, #9255), JNK (dilution, 1:1000; Cell Signalling Technology, #9252), p‐MAP4K4 (dilution, 1:1000; LMAI Bio, #LM‐5493R), MAP4K4 (dilution, 1:1000; FineTest, #FNab04983), phosphorylated NF‐κB p65 (P‐p65, dilution, 1:1000; Cell Signalling Technology, #3039), p65 (dilution, 1:1000; Cell Signalling Technology, #8242), p‐IκBα (Ser32) (dilution, 1:1000; Cell Signalling Technology, #2859), IκBα (dilution, 1:1000; Cell Signalling Technology, #9242), phosphorylated p38‐mitogen‐activated protein kinases (P‐p38MAPK, 1:2000; Cell Signalling Technology, #9216), p38MAPK (dilution, 1:1000; Cell Signalling Technology, #8690), phosphorylated extracellular signal‐regulated kinase (P‐ERK, 1:1000; Cell Signalling Technology, #4376), ERK (dilution, 1:1000; Cell Signalling Technology, #4695), lamin A/C (dilution, 1:3000; Cell Signalling Technology, #4777), P‐PKCθ (Ser643/676) (dilution, 1:1000; Cell Signalling Technology, #9376), PKCθ (dilution, 1:1000; Cell Signalling Technology, #13643), P‐PKCα (Thr638) (dilution, 1:1000; Cell Signalling Technology, #9375), PKCα (dilution, 1:1000; Cell Signalling Technology, #2056), P‐PKCβ (Ser660) (dilution, 1:1000; Cell Signalling Technology, #9371), PKCβ (dilution, 1:1000; Cell Signalling Technology, #46809), P‐PKCλ (Thr403) (dilution, 1:1000; Cell Signalling Technology, #9378), PKCλ (dilution, 1:1000; Cell Signalling Technology, #2998) and glyceraldehyde 3‐phosphate dehydrogenase (GAPDH,1:3000; Cell Signalling Technology, #5174). GAPDH was used for normalisation. Secondary antibodies were peroxidase affinipure goat anti‐rabbit‐IgG (1:3000; Jackson ImmunoResearch Laboratories, USA, #111‐035‐003) and goat anti‐mouse‐IgG (1:3000; Jackson ImmunoResearch Laboratories, USA, #115‐035‐146). The WB bands were visualised using an enhanced chemiluminescence kit (PerkinElmer Inc., Waltham, MA, USA).

### 2.16 Luciferase Assay

The NF‐κB luciferase reporter plasmid was constructed by HanBio Therapeutics (Shanghai, China). MAECs were transfected with luciferase reporter plasmids or control plasmids with jetPEI transfection reagent (Polyplus‐transfection SA, Illkirch‐Graffenstaden, France). After 24 h, the cells were treated with sweroside at 1 μg/mL for 24 h, followed by PA at 0.4 mmol/L for an additional 24 h. Renilla luciferase activity was assessed with a dual‐luciferase reporter assay (Promega Corporation, Madison, WI, USA) in accordance with the manufacturer's instructions.

### 2.17 Blood Pressures and Other Parameters

Blood pressures, including systolic blood pressure and diastolic blood pressure, were noninvasively measured by the tail‐cuff method (Softron BP‐98A, Tokyo, Japan). Blood pressure values were averaged from three consecutive measurements under steady‐state conditions. Food intake, faecal output and lipid content in faeces were measured as previously described [20, 21].

### 2.18 Chromatin Immunoprecipitation (ChIP) Assay

MAECs were transfected with siMAP4K4 or siCon. After 12 h, the cells were treated with sweroside at 1 μg/mL for 24 h, followed by PA at 0.4 mM for an additional 24 h. The ChIP assay was performed using the SimpleChIP enzymatic chromatin IP kit (Cell Signalling Technology, #9003) in accordance with the manufacturer's instructions with Abs targeting p65 (Cell Signalling Technology, #8242) and IgG (Cell Signalling Technology, #2729). The primers for detection of IKBα, VCAM‐1 and E‐selectin are listed in Table S1. The target genes were amplified by RT‐PCR.

### 2.19 Mouse Biochemical Measurements

Blood samples were obtained from mice after a 12‐h fast and centrifuged. Serum samples were stored at −80°C until further analysis. TC, TG, LDL‐C, HDL‐C, HbA1c and creatinine concentrations were measured with commercial colourimetric assay kits (Nanjing Jiancheng Bioengineering Institute). Fasting blood glucose (FBG) and postprandial 2‐h blood glucose (after 75‐g glucose loading) were measured with the glucose oxidase assay. Serum fasting insulin levels were measured with an electrochemiluminescence immunoassay. FFA levels were measured using an enzymatic colourimetric assay (Roche Diagnostics, Mannheim, Germany) in accordance with the manufacturer's instructions. Serum levels of TNF‐α, IL‐1β, IL‐6, ICAM‐1, VCAM‐1 and E‐selectin were measured using ELISA kits (R&D Systems Inc.). The coefficients of variation of these assays were 1% to 2% (blood glucose, HDL‐C and TNF‐α), 2% to 3% (HbA1c, TC and IL‐1β) and 3% to 6% (insulin, LDL‐C, FFA, TG and IL‐6).

### 2.20 Statistical Analysis

All data are presented as the means ± standard error of the mean (SEM). The unpaired Student's *t*‐test was used for comparisons of data between two groups and one‐way ANOVA followed by Tukey's post hoc test for comparisons of data among multiple groups. A probability (*p*) value < 0.05 was considered statistically significant.

## 3 Results

### 3.1 Sweroside Alleviated Endothelial Injury, AS and Inflammation in AKO Mice

**FIGURE 1:**
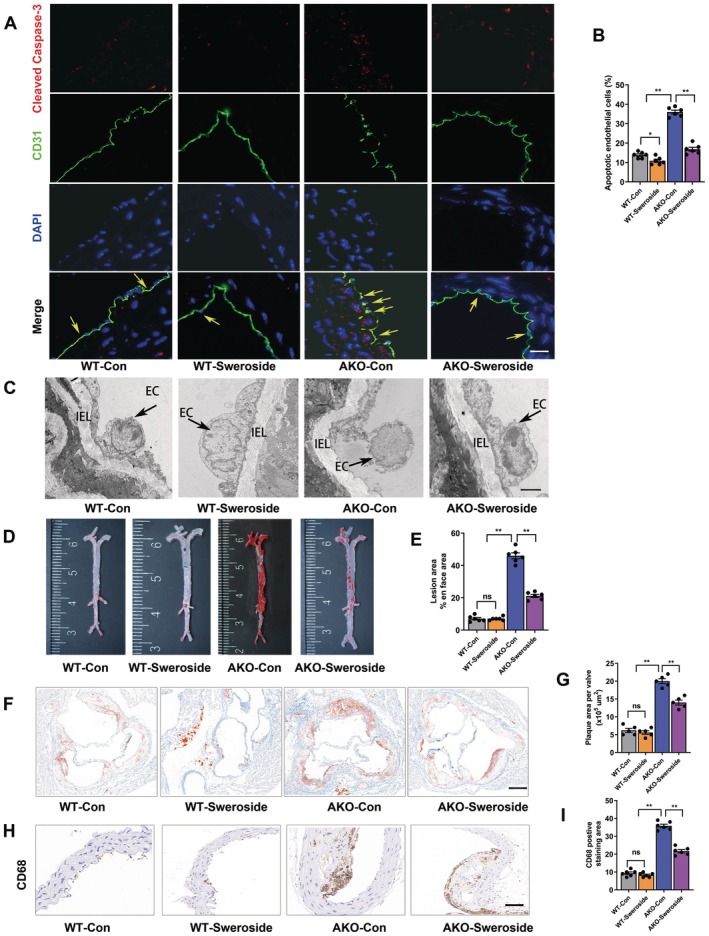
Sweroside alleviated endothelial injury and atherosclerosis. Treated with sweroside in WT and AKO mice aged 4 to 6 weeks. Then, the mice were fed a WD for 12 weeks, and atherosclerosis was assessed at the end of the experiment. (A) Representative images of Cleaved Caspase‐3 and CD31 double staining in sections of thoracic aortas. Green fluorescence represents CD31, red fluorescence represents Cleaved Caspase‐3 and blue fluorescence represents DAPI. Scale bars, 200 μm. (B) The percentage of apoptotic endothelial cells (*n* = 6). (C) Representative electron microscopy images of endothelium. Scale bars, 50 μm. (D) Representative images of en face atherosclerotic lesions. (E) Quantitative analysis of (D) (*n* = 6). (F) Representative images of the cross‐sectional area of the aortic root. Scale bars, 200 μm. (G) Quantitative analysis of (F) (*n* = 5). (H) Representative immunohistochemical staining images of CD68 in aortic plaques. Scale bar, 100 μm. The black arrows show aortic plaques. (I) Quantitative analysis of (I) (*n* = 6). The data are shown as the means ± SEM. *p < 0.05, **p < 0.01. Significant differences were determined by Student's *t*‐test or one‐way ANOVA followed by Tukey's post‐test.

Models of WD‐induced AS in mice were successfully established. At the end of the three‐month period, the effects of sweroside on endothelial injury and inflammation responses were investigated in AKO mice. It is well‐known that inflammation accelerates endothelial injury [22]. To verify that sweroside inhibited inflammation and adhesion responses, as well as attenuated endothelial injury and AS in AKO mice, the mice were intragastrically administered sweroside. Next, further experiments with WT + saline control (WT‐Con), WT + sweroside (WT‐Sweroside), AKO + saline control (AKO‐Con) and AKO + sweroside (AKO‐Sweroside) groups were conducted. All mice were fed with WD for 12 weeks. The results showed that sweroside decreased apoptosis of ECs (Figure 1A–C), reduced inflammation and adhesion molecule expression of MAECs, improved insulin resistance (IR), inflammation and decreased body weight gain (Table S2, Figure S1A–H) as compared to AKO mice. These results suggested that sweroside alleviated inflammation and endothelial injury.

Next, to determine whether sweroside blunted AS. As expected, sweroside treatment reduced the areas of atherosclerotic lesions and improved cellular components within atherosclerotic plaques in WD‐fed AKO mice (Figure 1D–I) as compared to the control group. These results confirmed that sweroside attenuated AS.

### 3.2 Sweroside Attenuated Leukocyte Homing in the Aortas of AKO Mice

**FIGURE 2:**
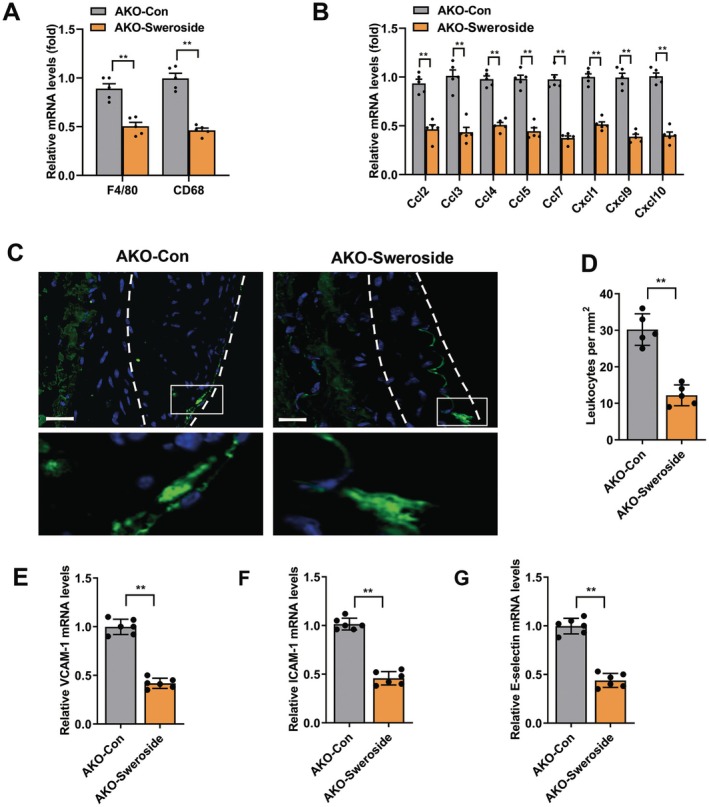
Sweroside decreased the leukocytes homing in aortic plaques from AKO mice. Sweroside was treated in AKO mice aged 4 weeks, and leukocyte homing was analysed in AKO‐con or AKO‐Sweroside mice that had been fed a WD for 12 weeks. (A) The mRNA expression of the macrophage markers F4/80 and CD68 in aortas (*n* = 5). (B) The mRNA expression of the chemokines in aortas (*n* = 5). (C, D) The homing of GFP leukocytes to atherosclerotic plaques 48 h after injection into AKO‐con or AKO‐Sweroside mice that were fed a WD for 12 weeks. (C) Fluorescence micrograph of aortic root plaques. The dashed line indicates the plaque border. Inset, magnification of GFP leukocytes. Blue, DAPI; Green, GFP. Scale bars, 150 μm. (D) Quantification of GFP leukocytes per square millimetre of plaque (*n* = 5). (E–G) The mRNA expressions from MAECs of aorta for VCAM‐1 (E), ICAM‐1 (F) and E‐selectin (G) (*n* = 6). Data were shown as means ± SEM. **p < 0.01. Significant differences were determined by Student's *t*‐test.

Inflammation induces leukocyte homing and macrophage accumulation within aortic plaques [5]. Thus, leukocyte recruitment after sweroside treatment was investigated in AKO mice fed a WD for 12 weeks. As shown in Figure 2A,B, mRNA expression levels of macrophage marker genes (F4/80 and CD68) and endothelial‐derived chemokines, which contribute to leukocyte homing, were decreased in the aortas of mice in the AKO‐Sweroside group as compared to the AKO‐Con group. Next, thioglycollate‐stimulated peritoneal exudate cells were extracted from GFP‐positive mice and injected intravenously into AKO‐Sweroside and AKO‐Con mice. Then, GFP‐positive cell levels of the aortic roots were quantified to assess leukocyte homing (Figure 2C). As compared to AKO‐Con mice, GFP‐positive cells within plaques were reduced by 65% in AKO‐Sweroside mice (Figure 2D). The leukocyte adhesion molecules ICAM‐1 and VCAM‐1 are required to mediate leukocyte homing in response to endothelial injury [5]. The mRNA expression levels of VCAM‐1, ICAM‐1 and E‐selectin in MAECs of the aorta showed similar changes after being treated with sweroside (Figure 2E–G). These results demonstrated that sweroside inhibited endothelial adhesion responses and alleviated leukocyte homing and macrophage accumulation of atherosclerotic plaques.

### 3.3 Sweroside Reduced PA‐Induced Apoptosis, Permeability, Inflammation and Oxidation of MAECs

**FIGURE 3:**
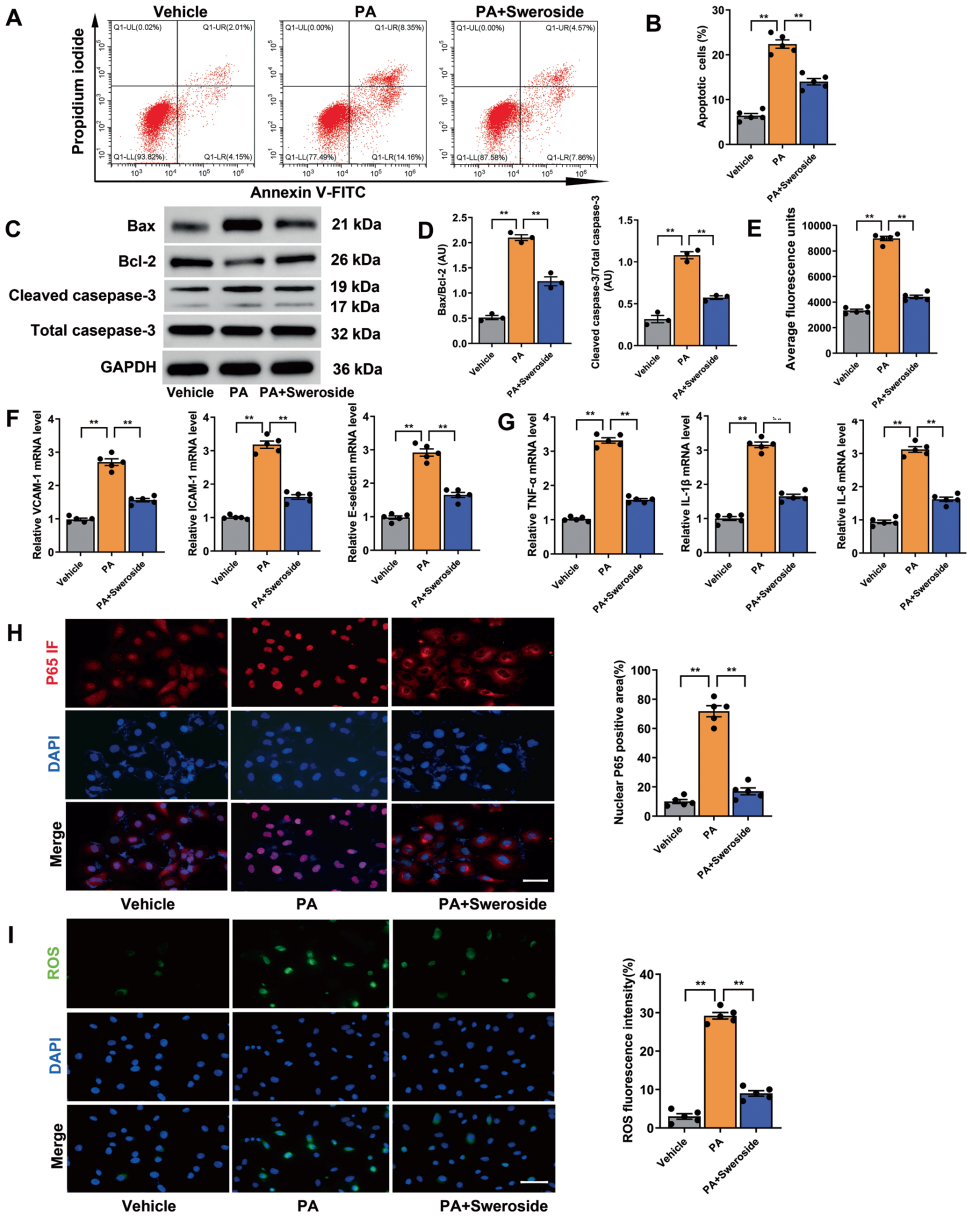
Sweroside reduced apoptosis, permeability, oxidation and inflammatory responses in MAECs induced by PA. The MAECs from WT mice were pretreated with 1 μg/mL sweroside for 24 h and 0.4 mmol/L PA for 24 h. (A) Representative images of apoptotic cells by flow cytometry assay in MAECs. (B) The percentage analysis of apoptotic cells. (C) The representative images of expressions for bax, bcl‐2 and cleaved caspase‐3 in MAECs by western blot. (D) Quantitative analysis of C. (E) MEACs permeability. (F) The levels of adhesion molecules and (G) inflammatory cytokines mRNA levels in lysate of MAECs. (H) The p65 nuclear translocation in MAECs. Scale bars, 50 μm. Red fluorescence represents p65 and blue fluorescence represents DAPI. (I) Representative images of ROS staining in MAECs. Scale bar, 50 μm. Green fluorescence represents ROS and blue fluorescence represents DAPI. Each experiment repeated 5 times. Data were shown as means ± SEM. **p < 0.01. Significant differences were determined by Student's *t*‐test or one‐way ANOVA followed by Tukey's post‐test.

To assess the direct effect of sweroside on the endothelium in vitro, MAECs were treated with sweroside. PA was used to induce endothelial injury for the in vitro experiments, and PA treatment at 0.4 mM for 24 h was selected for the following experiments [15]. As shown in Figure S2A, treatment with sweroside at 1 μg/mL for 24 h was optimal for the proliferation of MAECs. The experimental results showed that treatment with 1 μg/mL sweroside for 24 h increased the proliferation and migration of MAECs as compared to treatment with the vehicle only (Figure S2B–E). As compared to treatment with the vehicle alone, sweroside treatment attenuated apoptosis of MAECs by decreasing the expression of pro‐apoptotic proteins (Cleaved caspase‐3 and bax) and increasing the expression of the anti‐apoptotic protein bcl‐2, in addition to decreasing permeability of the endothelium and reducing the expression of molecules associated with inflammation (TNF‐α, IL‐6 and IL‐1β) and adhesion (ICAM‐1, VCAM‐1 and E‐selectin), as well as decreasing nuclear translocation of p‐p65 and increasing anti‐oxidant activity (Figure 3A–I). Collectively, these results showed that sweroside induced proliferation and reduced apoptosis, permeability, inflammation, and oxidation of PA‐treated MAECs.

### 3.4 MAP4K4/NF‐κB Signalling Is Essential for the Effects of Sweroside Against AS

**FIGURE 4:**
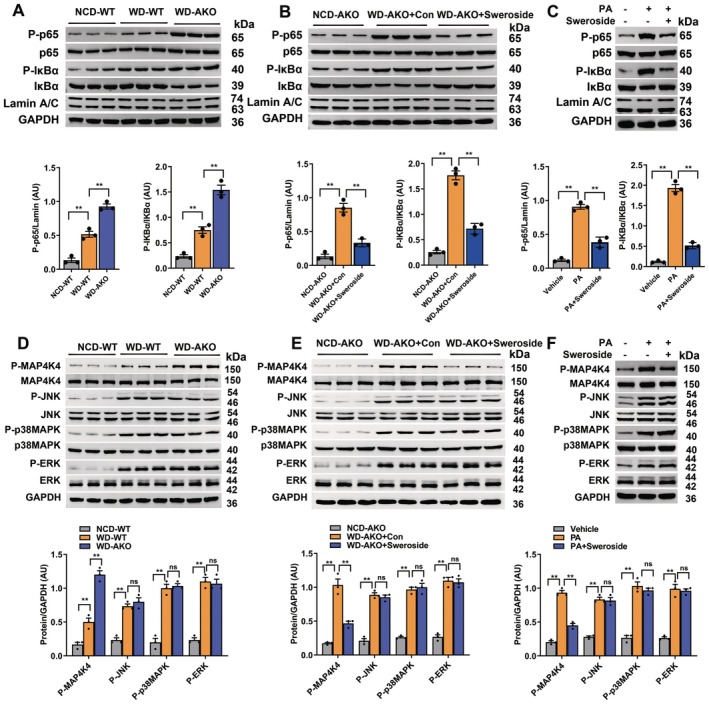
Sweroside inhibits MAP4K4/NF‐κB signalling in vivo and in vitro. (A) The levels of NF‐κB signalling proteins expressed in the MAECs of WT and AKO mice after 12 weeks of intervention (*n* = 6). (B) The levels of NF‐κB signalling proteins expressed in MAECs of AKO mice 12 weeks after sweroside treatment (*n* = 6). (C) The levels of NF‐κB signalling proteins expressed in MAECs from WT mice after sweroside treatment for 24 h or PA treatment for 24 h. (D) The upstream proteins expression of NF‐κB signalling MAECs of in WT and KO mice after 12 weeks of intervention (*n* = 6). (E) The upstream proteins expressed in NF‐κB signalling in MAECs of AKO mice after sweroside treatment for 12 weeks (*n* = 6). (F) The upstream proteins expressed in NF‐κB signalling in MAECs from WT mice after sweroside treatment for 24 h or PA treatment for 16 h. GAPDH was used as a loading control. The data are shown as the means ± SEM. Each in vitro experiment was repeated 5 times. **p < 0.01. Significant differences were determined by Student's *t*‐test or one‐way ANOVA followed by Tukey's post‐test.

AS is a chronic inflammatory disease and activation of NF‐κB signalling contributes to inflammatory reactions [6, 23]. Thus, the mechanisms underlying the protective effects of sweroside against AS were investigated. The above results showed that sweroside inhibited inflammation of the endothelium and the accumulation of macrophages in aortic plaques. In addition, the expression levels of p‐IκB‐α and nuclear p‐p65 were increased in MAECs of AKO mice as compared to WT mice (Figure 4A), but reduced in sweroside‐treated mice (Figure 4B). In addition, pretreatment with sweroside inhibited PA‐induced upregulation of p‐IκBα and nuclear p‐p65 in MAECs (Figure 4C). These results confirmed that NF‐κB signalling was at least partially involved in the beneficial effects of sweroside against endothelial inflammation.

NF‐κB signalling is regulated by JNK, p38MAPK, ERK and MAP4K4 [6, 7]. Expression of p‐MAP4K4 was increased in the MAECs of AKO mice as compared to WT mice (Figure 4D), but decreased in sweroside treated mice, while the expression levels of p‐JNK, p‐p38MAPK and p‐ERK were not affected by sweroside (Figure 4E). Similarly, pre‐treatment with sweroside inhibited PA‐induced p‐MAP4K4 expression in MAECs, but had no effect on the expression levels of p‐JNK, p‐p38MAPK and p‐ERK (Figure 4F). These data suggested that MAP4K4 signalling may be involved in the modulation of NF‐κB signalling.

**FIGURE 5:**
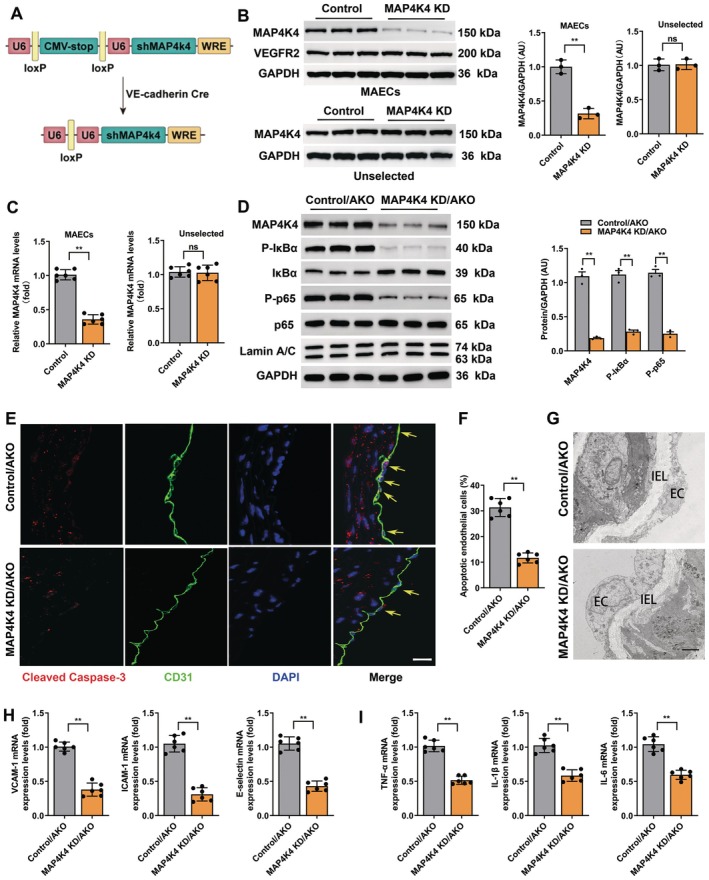
Endothelial‐specific MAP4K4 KD vitiates sweroside‐mediated protection of endothelial injury in vivo. MAP4K4 KD/AKO and control/AKO mice were fed a WD for 12 weeks, and endothelial injury and NF‐κB signalling were investigated. (A) Schematic of the transgenic construct used to generate MAP4K4 KD animals. (B, C) MAECs were derived from MAP4K4 KD and control mice. (B) MAP4K4 and vascular endothelial growth factor receptor (VEGFR2) protein expression in MAECs and unselected cell lysates (*n* = 3). (C) mRNA levels of MAP4K4 in immune‐selected or unselected cells (*n* = 6). (U6, promoter; WRE, woodchuck hepatitis virus posttranscriptional regulatory element). (D) The expression levels of NF‐κB signalling in MAECs of MAP4K4 KD/AKO and control/AKO mice (*n* = 3). Glyceraldehyde‐3‐phosphate dehydrogenase (GAPDH) was used as a loading control. (E) Representative images of Cleaved Caspase‐3 and CD31 double staining in sections of thoracic aortas. Green fluorescence represents CD31, red fluorescence represents Cleaved Caspase‐3 and blue fluorescence represents DAPI. Scale bars, 200 μm. (F) The percentage of apoptotic endothelial cells (*n* = 6). (G) Representative electron microscopy images of endothelium. Scale bars, 50 μm. (H) The mRNA levels of adhesion molecules and (I) inflammation in MAECs of aortas (*n* = 6). The data are shown as the means ± SEM. **p < 0.01. Significant differences were determined by Student's *t*‐test or one‐way ANOVA followed by Tukey's post‐test.

Next, to confirm the effects of MAP4K4 signalling in vivo, mice with endothelial‐specific KD of MAP4K4 (MAP4K4 shRNA‐VE‐cadherin Cre‐expressing mouse, MAP4K4 KD) were generated (Figure 5A) [5, 15]. In MAECs of MAP4K4 KD mice, the mRNA and protein expression levels of MAP4K4 were significantly reduced as compared to control MAECs (no Cre expression) transfected with shRNA targeting MAP4K4 (Figure 5B,C), confirming endothelial‐specific KD of MAP4K4. Then, male MAP4K4 KD/AKO and control/AKO mice were fed a WD for 12 weeks. Initially, the MAP4K4 KD/AKO mice were confirmed (Figure S3A,B). However, the results of the formal experiments showed that protein levels of p‐IκBα and nuclear p‐p65 were decreased in MAECs of MAP4K4 KD/AKO mice (Figure 5D). Consequently, endothelial injury and endothelial inflammation were improved in MAP4K4 KD/AKO mice as compared to the control/AKO mice (Figure 5E–I). These data confirmed that MAP4K4/NF‐κB signalling was at least partially essential for the beneficial effects of sweroside in the endothelium.

**FIGURE 6:**
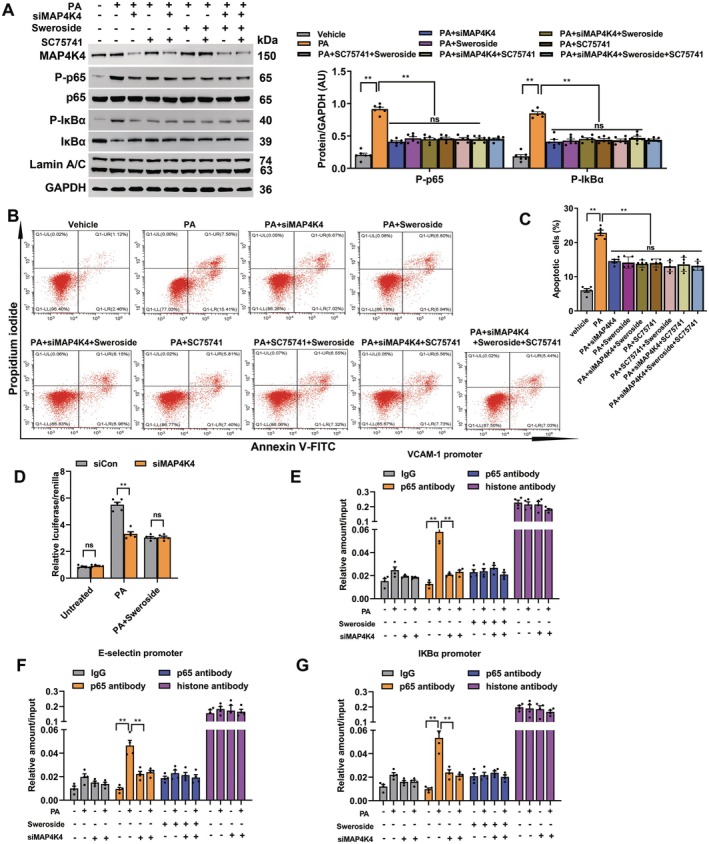
The results of signal proteins expression, luciferase and ChIP under MAP4K4 silencing in MAECs. (A–C) MAECs from WT mice were treated with siMAP4K4 for 60 h, sweroside for 24 h, SC75741 for 24 h, or PA for 24 h. (A) The levels of IkBα, P‐IkBα, p65 and P‐p65 expressions in MAECs induced by PA. (B) Representative images of apoptotic cells in MAECs. (C) The percentage analysis of apoptotic cells. (D) NF‐κB‐luciferase and SV40‐Renilla were transfected into MAECs after treatment with siCon or siMAP4K4. Cells were left untreated or treated with sweroside for 24 h, or/and PA for 24 h before luciferase and Renilla assessment. (E, F) MAECs from WT mice were transfected with siCon and siMAP4K4, then treated or not with sweroside for 24 h, or/and PA for 24 h. IgG, p65 and histone antibodies were used to immunoprecipitate chromatin and RT‐PCR was performed to determine (E) VCAM‐1, (F) E‐selectin and (G) IkBα promoters. The data represent the means ± SEM as normalised to input. **p < 0.01. Each experiment repeated 5 times. Significant differences were determined by Student's *t*‐test or one‐way ANOVA followed by Tukey's post‐test.

In addition, silencing of MAP4K4 in MAECs was performed in vitro (Figure S3C,D). The results showed increased expression of markers associated with inflammation (TNF‐α, IL‐1β and IL‐6) and adhesion (VCAM‐1, ICAM‐1 and E‐selectin), increased movement of fluorescein isothiocyanate‐labelled dextran across a monolayer, and monocyte adhesion to an activated endothelial monolayer in PA‐induced MAECs, which were diminished by silencing of MAP4K4. Furthermore, sweroside mimicked the roles of siMAP4K4 (Figure S3E–H), indicating that sweroside inhibited inflammation and monocyte adhesion to the endothelium at least partially through MAP4K4 in vitro. In addition, increased expression of NF‐κB in PA‐induced MAECs was reduced by silencing of MAP4K4 or in response to sweroside (Figure 6A). Consequently, increased PA‐induced apoptosis was attenuated by siMAP4K4 or sweroside intervention in MAECs (Figure 6B,C), indicating that sweroside inhibited NF‐κB through MAP4K4. Also, a luciferase reporter assay with NF‐κB‐binding elements of MAP4K4‐silenced MAECs was performed as reported previously [15]. The results showed that PA‐induced NF‐κB transcriptional activity was reduced by silencing of MAP4K4 and sweroside mimicked the effects of siMAP4K4 on NF‐κB transcriptional activity (Figure 6D). Furthermore, the results of the ChIP assay revealed that increased binding of p65 to the promoters of VCAM‐1, E‐selectin and IκBα induced by PA was decreased in MAECs by silencing of MAP4K4 and sweroside mimicked the roles of siMAP4K4 on p65 binding, indicating that sweroside inhibited NF‐κB transcriptional and binding activities at least partially through MAP4K4 (Figure 6E–G). Last, the NF‐κB inhibitor SC75741 (5 μM for 24 h) mimicked the effects of siMAP4K4 intervention in PA‐induced MAECs (Figures 6A–C and S3I), indicating that NF‐κB was a downstream target of MAP4K4. Together, these data suggested that endothelial MAP4K4/NF‐κB signalling at least partially was essential for the beneficial effects of sweroside against AS.

**FIGURE 7:**
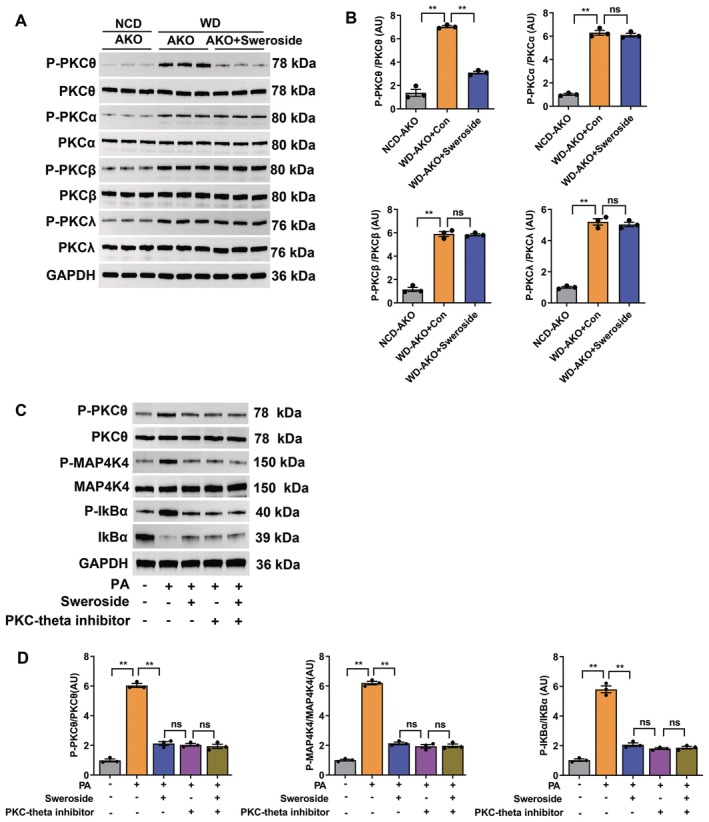
PKCθ is involved in the beneficial effects of sweroside that regulates the phosphorylation of MAP4K4 in MAECs. (A, B) The levels of PKC signalling proteins in sweroside‐treated MAECs of AKO mice (*n* = 3). (C, D) The levels of PKC signalling proteins in sweroside‐treated or PKCθ inhibitor‐treated MAECs. MAECs from WT mice were treated with sweroside for 24 h, PKCθ inhibitor for 24 h, or PA for 24 h. The data are shown as the means ± SEM. **p < 0.01. Significant differences were determined by Student's *t*‐test or one‐way ANOVA followed by Tukey's post‐test.

**FIGURE 8:**
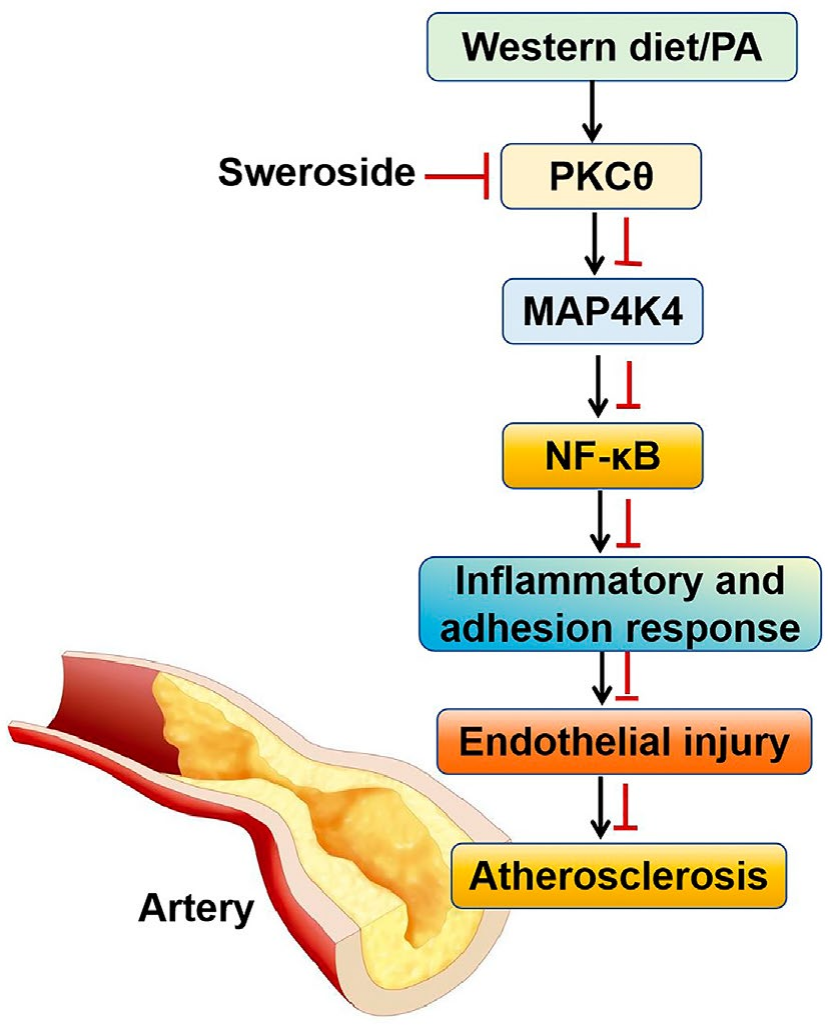
Schematic diagram of sweroside intervention in AS.

In the last experiments, we sought to explore how sweroside regulates the phosphorylation of MAP4K4 in MAECs. It is established that MAP4K4 is usually regulated by protein kinase C (PKC) [24, 25]. Thus, we determined the expression of PKC isoforms including α, β, θ and λ. Results showed P‐PKCθ in the MAECs was significantly decreased in sweroside‐treated mice compared with control mice (Figure 7A,B). However, the expression of P‐PKCα, P‐PKCβ or P‐PKCλ was not affected by sweroside (Figure 7A,B). Besides, sweroside treatment in MAECs decreased the expression of P‐MAP4K4 and P‐IKBα (Figure 7C,D). In addition, to further verify whether PKCθ is involved in the upstream events of MAP4K4 signalling, we treated MAECs with the PKCθ inhibitor, the results showed that the effects of treatment with 2 μM PKCθ inhibitor for 24 h strongly mimicked those of sweroside intervention, as evidenced by the significantly decreased expression of P‐PKCθ, P‐MAP4K4 and P‐IKBα (Figure 7C,D). These data suggested that PKCθ is involved in the regulation effects of sweroside on the phosphorylation of MAP4K4 in MAECs (Figure 8).

## 4 Discussion

AS is an inflammatory disease that is initiated by lipid‐mediated vascular inflammation of the vessel walls, which promotes continual monocyte recruitment that is dependent on leukocyte adhesion molecules [26, 27]. Therapeutic strategies for this disease include drugs or key cellular enzymes in endothelial cells that promote leukocyte attachment and permeability and may serve as drugs or their targets. Sweroside has antioxidant and anti‐inflammatory properties and has emerged as a potential agent to inhibit AS progression [10, 11]. Previous studies have shown that sweroside may have therapeutic effects on various inflammatory diseases such as nonalcoholic fatty liver disease and diabetic nephropathy [12, 13, 14]. We hypothesized that sweroside would ameliorate endothelial inflammation and adhesion responses and inhibit macrophage accumulation in plaques, and the data presented here support this hypothesis.

Endothelial injury is an early pathophysiological change in the development of AS [16]. Here, sweroside inhibited endothelial inflammation and adhesion responses, and reduced endothelial permeability and apoptosis induced by PA in vitro, suggesting that sweroside protected against endothelial injury. Aortic plaques are characterised by increased levels of macrophages and T lymphocytes and reduced levels of collagen and VSMCs [28, 29]. Next, we questioned whether sweroside alleviated late‐stage atherosclerotic lesions. We found that sweroside reduced the number of atherosclerotic plaques and ameliorated development of AS in AKO mice, as measured by histology of the aortic root and Oil Red‐O and CD68 staining of the aorta. Monocyte recruitment in a manner dependent on leukocyte adhesion molecules is crucial for the development of AS [30, 31]. Consistent with this, reduced macrophage marker and chemokine mRNA expression was observed in aortas from AKO animals when treated with sweroside. The cellular components of atherosclerotic plaques were improved and leukocyte homing to the plaques within the aortic arch was also markedly inhibited. The data indicated that sweroside attenuates AS and improves plaque components to support a stable plaque phenotype.

In parallel, sweroside treatment attenuated inflammation, monocyte adhesion, permeability and p65 nuclear translocation induced by PA in MAECs. These data indicated that sweroside improved the cellular components of atherosclerotic plaques and decreased macrophage accumulation within the plaques, thereby decreasing the number of plaques and attenuating AS. In accordance with our previous study [13, 14], sweroside improved IR and lipid profiles and decreased body weight gain. Thus, improved metabolic profiles also contributed to the anti‐atherosclerotic effects of sweroside.

It is important to address the possible pathways by which sweroside protects against AS. Many studies have revealed that endothelial NF‐κB is critical for the expression of leukocyte adhesion molecules, AS and macrophage homing to aortic plaques [5, 32], and pharmacological targeting of endothelial NF‐κB ameliorated leukocyte–endothelial cell interactions, as well as vascular permeability [32]. In addition to the inflammatory response, endothelial NF‐κB regulates systemic insulin sensitivity and even aging [32, 33]. Thus, the NF‐κB signalling pathway has been implicated in the pathogenesis of AS [34]. We confirmed that sweroside inhibited endothelial NF‐κB signalling, as evidenced by decreased endothelial inflammation, adhesion responses, macrophage accumulation in plaques and endothelial expression of p‐IκBα and nuclear p‐p65. In addition, MAP4K4, p38MAPK, ERK and JNK were identified as upstream targets of NF‐κB signalling [35, 36]. The results of animal studies showed that endothelial MAP4K4 at least partially was involved in the effect of sweroside on NF‐κB signalling, as confirmed by the in vitro experiments. However, sweroside had no effect on the expression profiles of other signalling molecules, including p38MAPK, ERK and JNK. In the present experiments we noted that MAP4K4 knockdown reduced the levels of p‐IκBα in WD‐induced AKO mice, indicating that upstream pathway activation was blocked, leading to reduced degradation of IκBα and hindering the transport of p65 to the nucleus. Consistently, our data showed that MAP4K4 knockdown reduced WD‐induced p65 nuclear localization and transcriptional activation. The detection of a decrease in p‐p65 levels indicated a reduction in the proportion or quantity of p65 molecules in an activated state, leading to a decrease in the transcriptional activation activity of p65 and a weakening of the output of the NF‐κB signalling pathway. The simultaneous low levels of p‐IκBα and p‐p65 strongly indicated that the NF‐κB signalling pathway was in a significantly inhibited state. KD of MAP4K4 in ECs resulted in inhibition of NF‐κB signalling and improved downstream events, whereas sweroside reversed these effects. Consistently, silencing of MAP4K4 partially reversed the increased activity of NF‐κB transcription and p65 binding induced by PA, while sweroside reversed these effects. Last, we also found that PKCθ is involved in the beneficial effects of sweroside that regulates the phosphorylation of MAP4K4 in MAECs. However, the underlying mechanisms by which sweroside modulates PKCθ are unclear and require further study. These data confirmed that endothelial MAP4K4/NF‐κB signalling was crucial for the beneficial effects of sweroside against AS.

These findings demonstrated that sweroside has potential as a clinical therapeutic agent for endothelial injury and AS by inhibiting endothelial inflammation and adhesion responses. However, it is necessary to fully consider its dose, safety and bioavailability in clinical applications. Our findings support the notion that targeting the MAP4K4/NF‐κB pathway in MAECs could be a viable approach for the treatment of inflammation‐induced AS. It has been reported that MAP4K4 promotes proinflammatory functions, and treatment of AKO mice with a small‐molecule MAP4K4 protein kinase inhibitor significantly reduced atherosclerotic plaque progression and promoted plaque regression [5]. All these data strongly support the idea that MAP4K4 may be a novel therapeutic target for the treatment of cardiovascular disease. One study reported that endothelial MAP4K4 KD mice did not display altered plasma lipid levels [5], while sweroside improved lipid profiles in our current and previous research [13, 14]. Thus, whether the effect of sweroside in alleviating AS is completely consistent with the action of MAP4K4 inhibitors or targeted compounds needs to be evaluated further.

There were some limitations to this study. First, although we showed that sweroside attenuated AS, the study was constrained by a small sample size and experiments conducted in a small AKO animal model. Expanding the research to include large animal models and large sample sizes would enhance the robustness of the conclusions. Second, whether the effect of sweroside in alleviating AS in ApoE KO mice can be used to alleviate the progression of AS in humans remains to be explored. Further studies of patients with AS are needed to explore the relationship between sweroside and the clinicopathological characteristics of patients with AS. Third, although we focused on the role of endothelial MAP4K4 using genetic models, MAP4K4 inhibition may have indirect effects on other signalling pathways within cells, so its potential off‐target effects need to be carefully evaluated in clinical applications. Lastly, body weight was reduced in sweroside‐treated AKO mice; however, the underlying mechanism remains unclear and needs to be further investigated, especially in adipose metabolism.

## 5 Conclusions

This study demonstrated that sweroside ameliorated endothelial inflammation and adhesion responses, inhibited macrophage accumulation in plaques, and alleviated endothelial injury and AS. The molecular mechanisms underlying these beneficial effects of sweroside at least partially involved the MAP4K4/NF‐κB signalling pathway. These results imply the possible translational value of this research into clinical settings in the future. Sweroside has therapeutic benefits in treating AS, endothelial injury and metabolic disorders; therefore, with safe doses and good bioavailability, sweroside may be developed in the future for the sake of better medical compliance. However, there is still a long way to go to bring this treatment to market because there are a lot of clinical trials and regulatory affairs to be completed and cost‐effectiveness analysis needs to be conducted.

## Author Contributions

**Minjiang Huang:** conceptualization (equal), investigation (equal), methodology (equal), writing – review and editing (equal). **Huiming Yin:** formal analysis (equal), methodology (equal), writing – review and editing (equal). **Qiansheng Yang:** software (equal), writing – review and editing (equal). **Lingli Liu:** software (equal), writing – review and editing (equal). **Yuefu Chen:** investigation (equal). **Ling Jin:** validation (equal), visualization (equal), writing – review and editing (equal). **Yang Yang:** data curation (equal), writing – original draft (equal), writing – review and editing (equal). **Ke Hu:** formal analysis (equal), project administration (equal). **Yan Ding:** conceptualization (equal), data curation (equal), investigation (equal), methodology (equal), project administration (equal), validation (equal), writing – review and editing (equal).

## Consent

The authors have nothing to report.

## Conflicts of Interest

The authors declare no conflicts of interest.

## Supporting information


**Data S1:** jcmm70837‐sup‐0001‐Supinfo.doc.

## Data Availability

The datasets generated and/or analysed during the current study are available from the corresponding author on reasonable request.
