# Supplementary material for: Sweroside Inhibits Inflammation and Alleviates Endothelial Injury and Atherosclerosis in Mice
Source: J Cell Mol Med. 2025 Sep 5;29(17):e70837. doi: 10.1111/jcmm.70837 (PMC12413309; doi:10.1111/jcmm.70837)
Supplement: Supplementary file 1 — Data S1: jcmm70837‐sup‐0001‐Supinfo.doc. [file JCMM-29-e70837-s001.doc]

**Sweroside inhibits inflammation and alleviates endothelial injury and atherosclerosis in mice**

Minjiang Huang, Huiming Yin, Qiansheng Yang, Lingli Liu, Yuefu Chen, Ling Jin, Yang Yang, Ke Hu,Yan Ding

**Table S1. A list of siRNA sequence and RT-PCR primers used in this study.**

|  | scrambled (5’ to 3’) | | siRNA (5’ to 3’) |
| --- | --- | --- | --- |
| MAP4K4 | 5’-CAGUCGCGUUUGCGACUGG-3’ | | 5’-GACCAACUCUGGCUUGUUA-3’ |
| Primer name | | Forward primer (5’ to 3’) | Reverse primer (5’ to 3’) |
| TNF-α | | AGCCCCCAGTCTGTATCCTT | CTCCCTTTGCAGAACTCAGG |
| IL-1β | | CCGTGGACCTTCCAGGATGA | GGGAACGTCACACACCAGCA |
| IL-6 | | ACTCCAGAAGACCAGAGGAAAT | CCAGAGATACAAAGAAATGATGG |
| MAP4K4 | | CATCTCCAGGGAAATCCTCAGG | TTCTGTAGTCGTAAGTGGCGTCTG |
| F4/80 | | CCCCAGTGTCCTTACAGAGTG | GTGCCCAGAGTGGATGTCT |
| CD68 | | CCATCCTTCACGATGACACCT | GGCAGGGTTATGAGTGACAGTT |
| ICAM-1 | | GTGATGCTCAGGTATCCATCCA | CACAGTTCTCAAAGCACAGCG |
| VCAM-1 | | AGTTGGGGATTCGGTTGTTCT | CCCCTCATTCCTTACCACCC |
| E-selectin | | ATGAAGCCAGTGCATACTGTC | CGGTGAATGTTTCAGATTGGAGT |
| GAPDH | | TCAACAGCAACTCCCACTCTTCCA | TTGTCATTGAGAGCAATG CCAGCC |
| IKBα  (ChIP) | | AAGCTGAGGCAGAGGTCCTA | GGGTGGTTTGAGACAGGGTT |
| VCAM-1  (ChIP) | | CCTGTCATCCAGCAATGGGTCA | AATGAAAGGACGTTGATGCAGAA |
| E-selectin (ChIP) | | GGCAGAGTCTCAGTTAAATAGTTGT | CCGCAGTTGCCTTGAACCT |

Abbreviations: TNF-α, tumor necrosis factor-α; IL-1β, interleukin-1β; IL-6, interleukin-6; MAP4K4, mitogen-activated protein kinase kinase kinase kinase 4; ICAM-1, intercellular adhesion molecule-1; VCAM-1, vascular cell adhesion molecule-1; GAPDH, glyceraldehyde 3-phosphate dehydrogenase; IKBα, I-kappa-B-alpha.

**Table S2. The metabolic and inflammatory characteristics in WT and AKO mice at terminal.**

| **Variables** | **WT-Con** | **WT-Sweroside** | **AKO-Con** | **AKO-Sweroside** |
| --- | --- | --- | --- | --- |
| N | 6 | 6 | 6 | 6 |
| FBG (mmol/L) | 5.07±0.12 | 4.87±0.23 | 5.07±0.08 | 5.25±0.12 |
| Insulin (mU/L) | 17.88±0.40 | 9.30±0.38^**^ | 20.65±0.57^**^ | 13.12±0.48^‡‡^ |
| HbAlc (%) | 5.28±0.15 | 5.15±0.10 | 5.18±0.11 | 5.45±0.11 |
| LDL-C (mmol/L) | 1.28±0.04 | 0.87±0.04^**^ | 2.07±0.04^**^ | 1.88±0.04^‡‡^ |
| HDL-C (mmol/L) | 1.17±0.03 | 1.49±0.03^**^ | 1.09±0.04^*^ | 1.30±0.03^‡‡^ |
| TG (mmol/L) | 1.21±0.04 | 0.92±0.03^**^ | 2.23±0.07^**^ | 1.32±0.02^‡‡^ |
| TC (mmol/L) | 3.43±0.06 | 3.05±0.07^**^ | 4.40±0.15^**^ | 3.53±0.03^‡‡^ |
| FFA (mmol/L) | 0.64±0.03 | 0.42±0.02^*^ | 1.06±0.04^**^ | 0.70±0.03^‡‡^ |
| SBP (mmHg) | 109.2±1.56 | 103.5±2.38 | 106.3±1.50 | 106.8±1.70 |
| DBP (mmHg) | 73.83±1.35 | 71.50±1.88 | 73.33±1.52 | 72.83±1.22 |
| TNF-α (pg/ml) | 38.77±0.96 | 20.78±0.52^**^ | 49.65±1.24^**^ | 26.03±1.67^‡‡^ |
| IL-1β (pg/ml) | 39.65±0.61 | 24.57±0.45^**^ | 50.67±0.76^*^ | 31.78±0.82^‡‡^ |
| IL-6 (pg/ml) | 27.15±0.93 | 12.82±0.53^**^ | 34.47±1.03^**^ | 19.17±0.57^‡‡^ |
| Body weight (g) | 27.88±0.29 | 26.50±0.25 | 34.83±0.34^**^ | 30.38±0.50^‡‡^ |
| Food intake (g/week) | 19.38±0.52 | 19.50±0.72 | 20.07±0.49 | 19.33±0.76 |
| Fecal output (g/week) | 2.28±0.08 | 2.22±0.09 | 2.18±0.07 | 2.35±0.07 |
| Lipid content in  feces (mg/g) | 21.02±0.74 | 20.83±0.79 | 39.90±0.5^**^ | 41.13±1.06^‡‡^ |
| VCAM-1(μg/ml) | 1.08±0.03 | 0.78±0.03^**^ | 1.27±0.06^**^ | 0.93±0.02^‡^ |
| ICAM-1(ng/ml) | 34.68±0.99 | 20.70±0.52^**^ | 41.27±0.61^**^ | 26.53±0.58^‡‡^ |
| E-selectin(ng/ml) | 51.17±0.92 | 35.13±0.74^**^ | 58.72±0.65^**^ | 44.03±0.82^‡‡^ |

Data are expressed as mean ± SEM. ^*^*P* < 0.05,^**^*P* < 0.01 vs. WT-Con; ^‡^*P* <0.05, ^‡‡^*P* <0.01 vs. AKO con. All mice were fed WD for 12 weeks. One-way ANOVA followed by Tukey’s post-test for multiple comparisons was used for groups of three or more.

Abbreviations: SBP, systolic blood pressure; DBP, diastolic blood pressure; FBG, fasting blood glucose; HbA1C, glycosylated hemoglobin; TG, triglycerides; TC, cholesterol; FFA, free fatty acid; TNF-α, tumor necrosis factor-α; IL-6, interleukin-6; LDL-C, low-density lipoprotein-cholesterol; HDL-C, high-density lipoprotein-cholest

erol; ICAM-1, intercellular adhesion molecule-1; VCAM-1, vascular cell adhesion molecule-1.


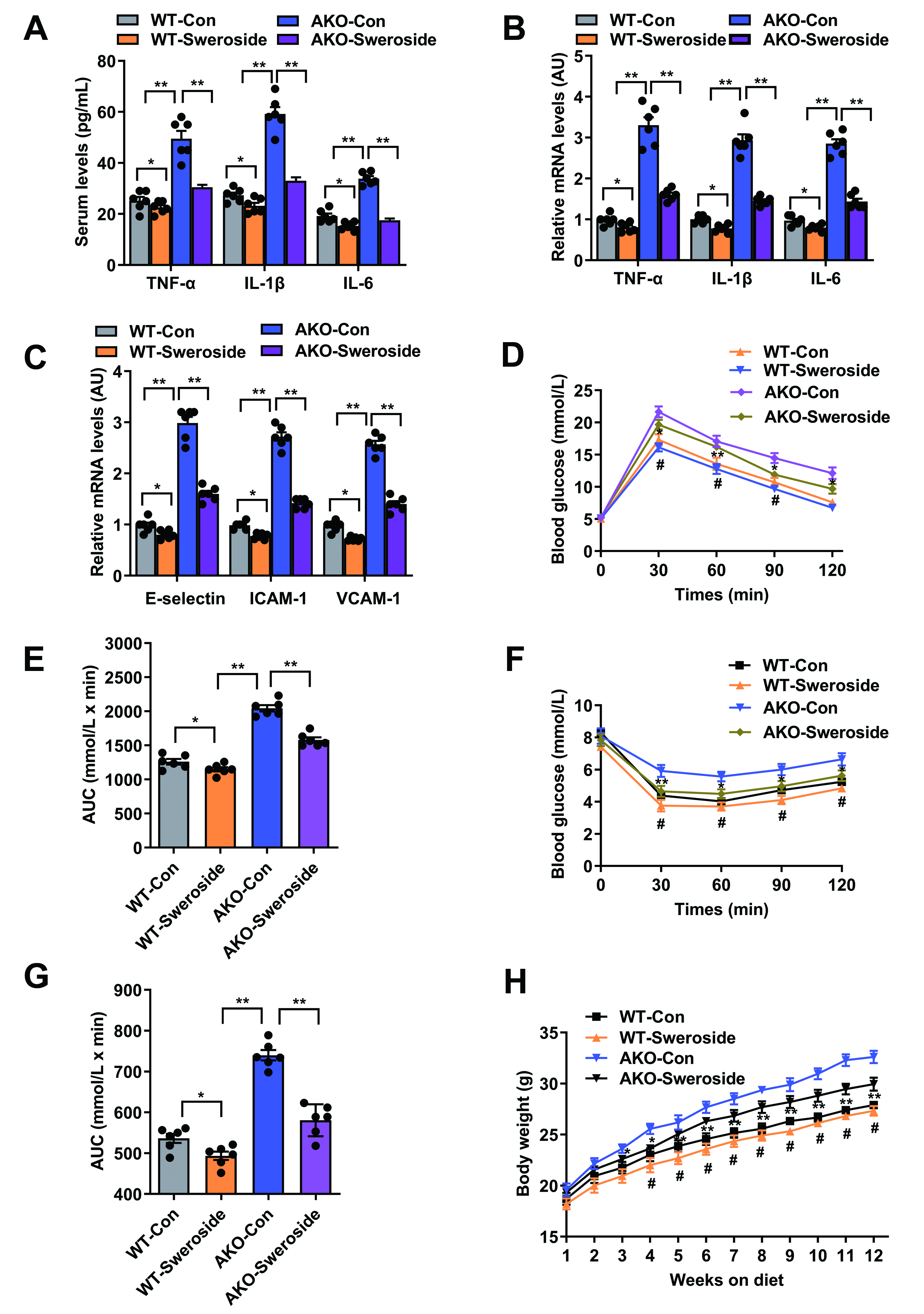


**Supplementary Figure 1. Sweroside attenuated inflammation and metabolic disorders in AKO mice.** The mice were fed a WD for 12 weeks and the inflammation and metabolic profiles were determined at the end (6 mice in each group). (A) The serum levels of TNF-α，IL-1β and IL-6 in different treated mice. (B)The mRNA levels of TNF-α，IL-1β and IL-6 in MAECs of aortas in different treated mice (n = 6). (C) The mRNA levels of adhesion molecules in MAECs of aortas (n=6). (D) Results of GTT (n=6). GTT was tested by a single i.p. injection of glucose (2 g/kg of body weight) in 12 hours-fasted mice at terminal.^*^*P* < 0.05, ^**^*P* < 0.01 versus AKO-Con. ^#^*P* < 0.05, ^##^*P* < 0.01 versus WT-Con. (E) The AUC for GTT using the trapezoidal rule. (F) Results of ITT (n=6). ITT was performed by a single i.p. injection of insulin (0. 75 U/kg of body weight) in 6 hours-fasted mice at terminal.^*^*P* < 0.05, ^**^*P* < 0.01 versus AKO-Con. ^#^*P* < 0.05, ^##^*P* < 0.01 versus WT-Con. (G) The AUC for ITT. (H) Body weight. ^*^*P* < 0.05, ^**^*P* < 0.01 versus AKO-Con. ^#^*P* < 0.05, ^##^*P* < 0.01 versus WT-Con.We recorded the body weight of each group weekly for 12 weeks (n=6). Data were shown as means ± SEM. ^*^*P* < 0.05, ^**^*P* < 0.01. Significant differences were determined by Student’s *t*-test.

**
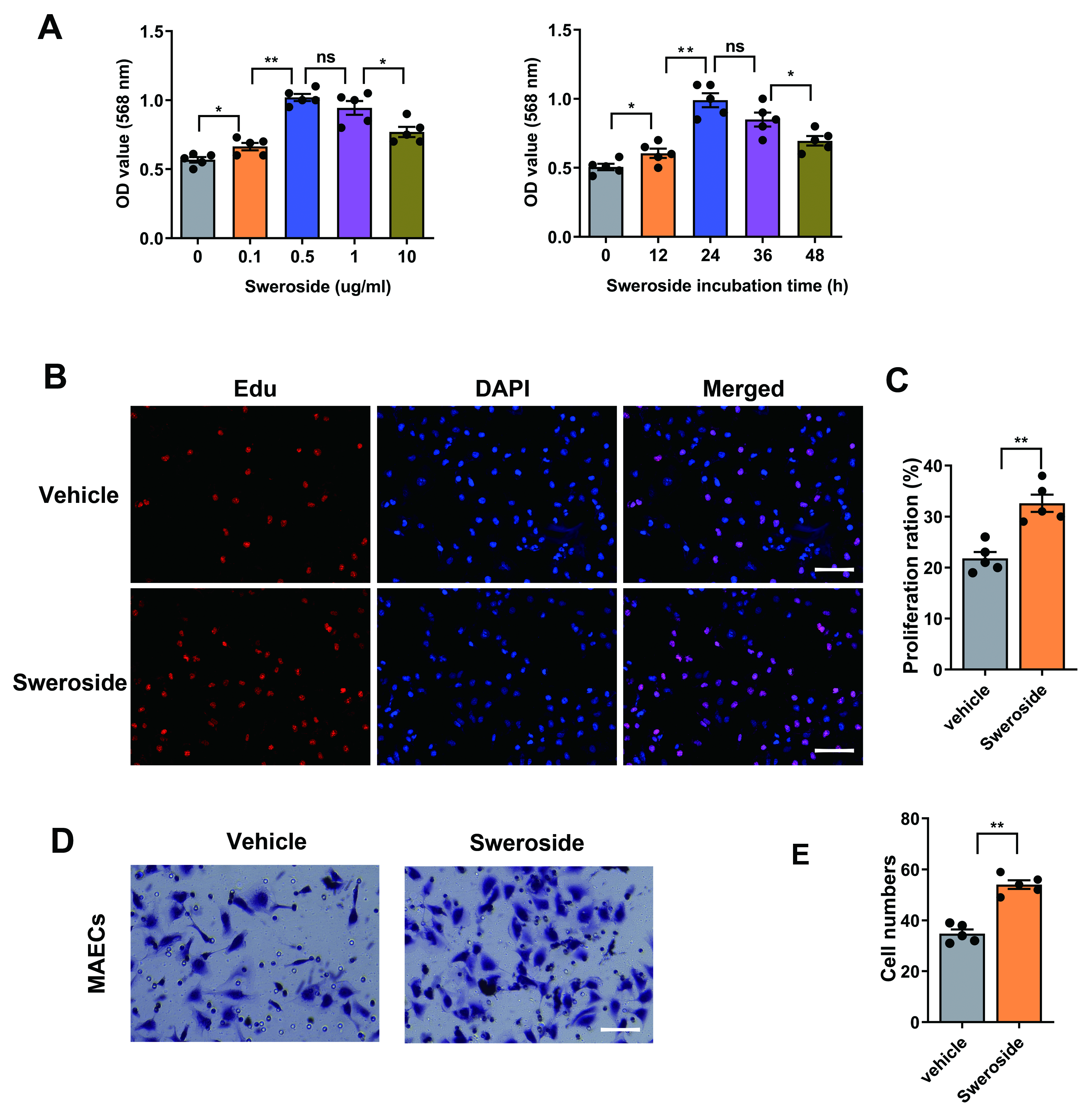
**

**Supplementary Figure 2. Identification of the optimum incubation conditions, proliferation and migration of MAECs.** (A) The MAECs (from WT mice) proliferation responses of time- and dose- dependent induced by sweroside. (B) Representative proliferation images of MAECs. Magnification×200. Scale bar, 200μm. (C) Quantitative analysis of B. (D) Representative migration images of MAECs. Magnification×200. Scale bar, 100μm. (E) Quantitative analysis of D. Data were shown as means ± SEM. Each experiment repeated 5 times. ^*^*P* < 0.05, ^**^*P* < 0.01. Significant differences were determined by Student’s *t*-test or one-way ANOVA followed by Tukey’s post-test.

**
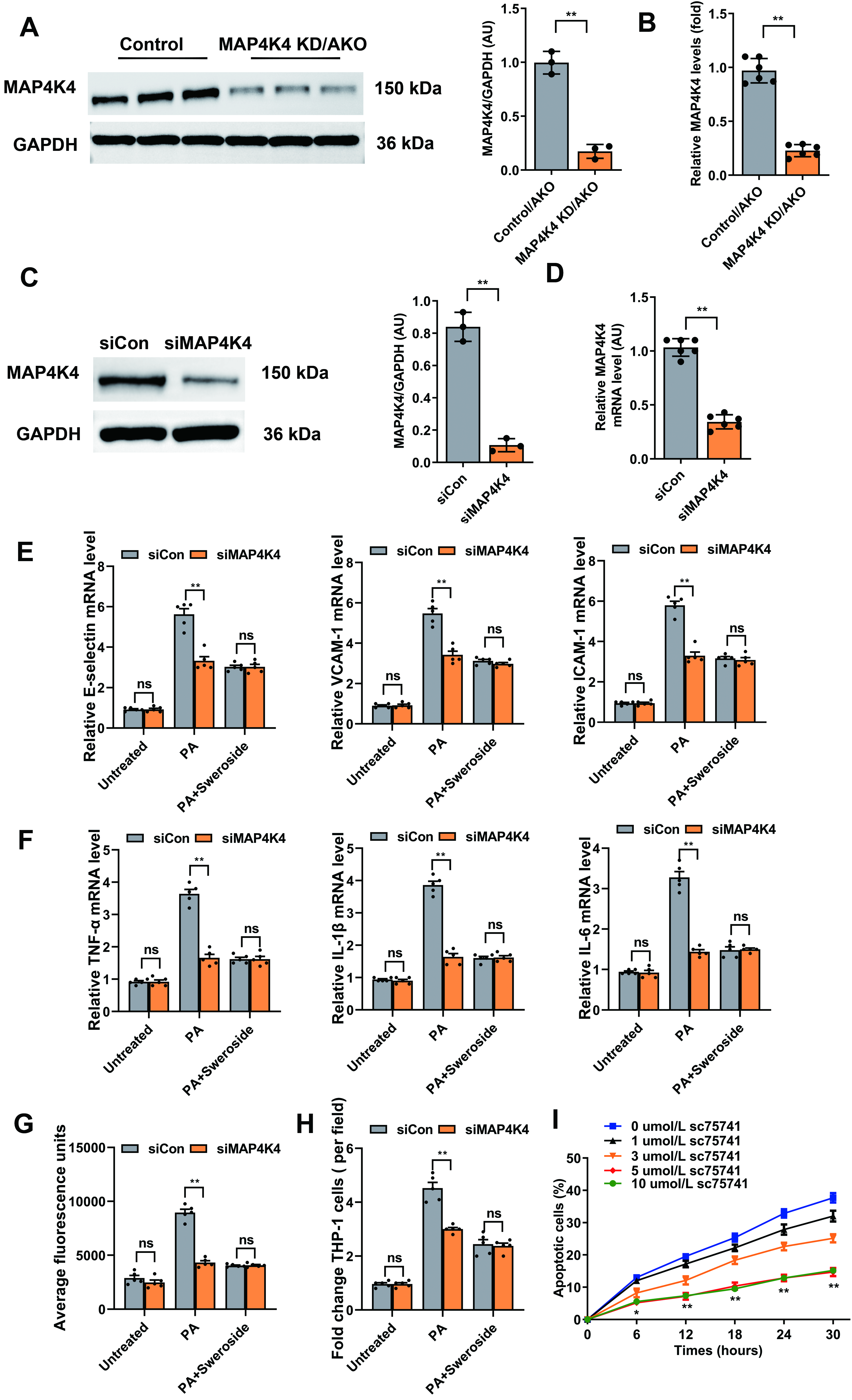
**

**Supplementary Figure 3. The confirmation of MAP4K4 knockdown in MAP4K4 KD/AKO mice or silencing in MAECs in vitro, as well as the adhesion and inflammatory responses after siCon and siMAP4K4 treatment in MAECs.** (A and B) MAP4K4 KD/AKO mice and control/AKO mice were fed a WD for 12 weeks, and MAECs were obtained at the end of experiments. (A) The MAP4K4 protein expression (n = 3) and (B) The MAP4K4 mRNA expression in MAECs from aortas (n = 6). (C) MAP4K4 protein levels in MAECs from WT mice after transfected siRNA for MAP4K4. (D) MAP4K4 mRNA levels in MAECs from WT mice after transfected siRNA for MAP4K4. (E-I) The MAECs were obtained from WT mice. The siCon and siMAP4K4 MAECs were treated with sweroside 1 μg/ml for 24 hours or PA 0.4mmol/L for 16 hours. The adhesion responses and inflammation were then determined. (E) The mRNA expressions of VCAM-1 and ICAM-1 in MAECs. (F) The mRNA expressions of TNF-α, IL-6 and IL-1β in MAECs. (G) Cells were seeded onto transwell chambers. Confluent cells were treated with sweroside or PA, and FITC-labelled dextran that migrated through the MAECs monolayer was measured. The data represent the mean fluorescence intensity ± SEM. (H) THP-1 monocytes were stained with calcein green and adhered to PA activated MAECs for 30 minutes. Fluorescence microscopy was used to measure the number of adherent THP-1 monocytes per microscopic field (x100). (I)Time- and dose-dependent changes in PA-induced MAECs apoptosis in response to the NF-κB inhibitor SC75741. The data represent the means ± SEM as normalized to the unstimulated control time point. Each *in vitro* experiment was repeated 5 times. Data are presented as the means ± SEM. ^*^*P* < 0.05, ^**^*P* < 0.01. Significant differences were determined by Student’s *t*-test or one-way ANOVA followed by Tukey’s post-test.
